# Research and Application of Ancient Chinese Pattern Restoration Based on Deep Convolutional Neural Network

**DOI:** 10.1155/2021/2691346

**Published:** 2021-12-10

**Authors:** Xuhui Fu

**Affiliations:** Culture and Art Management of Hunan University, Guangzhou 62399, China

## Abstract

In recent years, deep learning, as a very popular artificial intelligence method, can be said to be a small area in the field of image recognition. It is a type of machine learning, actually derived from artificial neural networks, and is a method used to learn the characteristics of sample data. It is a multilayer network, which can learn the information from the bottom to the top of the image through the multilayer network, so as to extract the characteristics of the sample, and then perform identification and classification. The purpose of deep learning is to make the machine have the same analytical and learning capabilities as the human brain. The ability of deep learning in data processing (including images) is unmatched by other methods, and its achievements in recent years have left other methods behind. This article comprehensively reviews the application research progress of deep convolutional neural networks in ancient Chinese pattern restoration and mainly focuses on the research based on deep convolutional neural networks. The main tasks are as follows: (1) a detailed and comprehensive introduction to the basic knowledge of deep convolutional neural and a summary of related algorithms along the three directions of text preprocessing, learning, and neural networks are provided. This article focuses on the related mechanism of traditional pattern repair based on deep convolutional neural network and analyzes the key structure and principle. (2) Research on image restoration models based on deep convolutional networks and adversarial neural networks is carried out. The model is mainly composed of four parts, namely, information masking, feature extraction, generating network, and discriminant network. The main functions of each part are independent and interdependent. (3) The method based on the deep convolutional neural network and the other two methods are tested on the same part of the Qinghai traditional embroidery image data set. From the final evaluation index of the experiment, the method in this paper has better evaluation index than the traditional image restoration method based on samples and the image restoration method based on deep learning. In addition, from the actual image restoration effect, the method in this paper has a better image restoration effect than the other two methods, and the restoration results produced are more in line with the habit of human observation with the naked eye.

## 1. Introduction

With the birth and development of the Internet, the pace of technological innovation has accelerated the increase in demand, and our lives are changing with each passing day, promoting new things to continue to emerge around people. High-tech applications have provided tremendous help to human society. The traditional pattern recovery technology is a process of intelligently filling and repairing the missing or damaged part of the traditional pattern information by using the pixel information and related previous information in the original traditional pattern image. Traditional mode restoration is the process of filling in the defect area of the image information in the traditional mode. The purpose is to use the traditional mode information defect to restore the image, so that the observer cannot perceive that the traditional mode image has defects or has been repaired. At the beginning of the 21st century, Bertamio and others put forward the concept of digital image printing at the computer graphics annual meeting held by the American Computer Association Computer Graphics Professional Group (ACM SIGGRAPH). The core idea of this concept is to use computer algorithms and simulate the manual repair process to repair part of the missing image content. Digital image restoration technology is to obtain the prior knowledge of the image by calculating the remaining information in the damaged image, thereby using related algorithms to effectively estimate and then fill in the missing part, so as to restore the high-quality original image, making it impossible for people to detect the restoration. However, convolutional neural networks have outstanding advantages in pattern restoration. Unlike patch-based methods, they retain the neighborhood relationship and spatial locality of the input in the potential high-level feature representation. The number of free parameters that can describe their shared weights does not depend on the input dimensions. Unlike patch-based methods, convolutional neural networks can retain the connections between neighborhoods and local characteristics of the space. Compared with the common fully connected depth structure, They have no difficulty in processing high-dimensional images of actual size. For traditional patterns, image restoration has a good effect, because the convolutional neural network is based on a shared convolution kernel to process high-dimensional data, such as traditional images. The deep convolutional neural network is used to realize the traditional pattern image restoration process, that is, using multiple hidden images, which is realized by layer-by-layer transfer.

## 2. Related Work

The pattern repair method based on deep confluence neural network is a method that has just appeared in the field of pattern repair in the past two or three years. Judging from the existing research results, the band representation method of some modes can also repair undiscovered modes after certain adjustments. Xie et al. believe that repairing defective patterns [1, 2] is more difficult. Because after the lack of patterns, it is impossible to know which parts of the missing area need to be repaired, the algorithm must independently determine how to repair the pixel information. Iizuka et al. used a fully turnaround neural network structure as a framework [3] and used the expanded integrator and double discrimination network proposed by improving the context encoder [4] to repair the network. They can repair any large irregularly shaped patterns in defective areas, but the pattern repair results require postprocessing to achieve the desired repair effect, which increases the cost of pattern repair and destroys the integrity of the network. On this basis, Portenier et al. proposed a rough and complete network structure and introduced a follow-up mechanism [5–7], which improved the repair effect to a certain extent, but there were still some problems. Aiming at solving the problem of unsatisfactory repair effect when the defect area is large, Wang et al. proposed to generate a multicolumn convolutional neural network structure [8], using different sizes of convolution kernels to fully extract features, and designed a new type of confidence-driven model reconstruction loss, using a large number of techniques to improve the model recovery quality, to achieve very good visual effect. However, when dealing with large data sets, the recovery effect is not ideal, especially when there are many objects or scene categories in the data set. The network parameters and the repair result structure are difficult to adapt [9]. Portenier et al. expanded the number of channels in the input mode and passed manual intervention information such as contour constraints and color constraints to the network model to achieve the purpose of restoring the results of the interference mode through preset conditions [10]. Dong et al. improved local outlier algorithm FWMIL-LOF based on multi-instance learning. The algorithm uses the MIL (multi-instance learning) framework, introduces a weight function describing the importance of the data in the conversion process of the sample package, and adjusts the weight function by defining a penalty strategy, thereby determining that the examples of different feature attributes are in the package [10–12]. Yao Xiaofeng and others designed laser image restoration technology based on deep neural network. The deep learning neural network is used to map the contour structure of the damaged area of the laser image, the damaged area of the laser image is segmented according to the curve fitting theory, the relevant information of the damaged area of the laser image is filled to complete the laser image repair, and a simulation test of laser image restoration was carried out [13–15]. Fan Xingang started by building a deep learning framework, applied the parallel architecture of convolutional neural networks, trained the network by alternating unsupervised and supervised learning to achieve super-resolution reconstruction of the target image, and finally verified the effect of image restoration through experiments [16, 17]. According to the change of the main gradient of the adjacent area of the pattern, the method preferentially generates the information of the curve part of the pattern structure. Pixels and curves with higher gradients and close to the repair area are preferentially used for pattern repair. In this way, better results can be produced compared to Rong guo East China and France [18, 19].

Due to the large number of hidden layers in the deep neural network model, it is generally considered difficult to conduct model training. In addition, many deep neural network models use the BP structure to train the model, which makes it suitable for solving problems in supervised learning, while the image repair problem is essentially biased towards unsupervised learning. Therefore, it is difficult to generate the information of the occluded part of the image using only the general deep neural network model.

## 3. Methodology

Deep convolution neural network (DCNN), as an important calculation model in deep learning theory, has extremely high processing efficiency and effects for traditional pattern images. In 2012, a model based on a deep convolutional neural network participated in the ImageNet Large Scale Visual Recognition Challenge 2012 (ILSVRC2012), reducing the error rate of the classification task from more than 26% to 15.32%. In the following years, the model based on the deep convolutional neural network has been continuously improved, and the classification error rate on the ImageNet data set has been declining. It has reached 4.94%, which is lower than the human eye recognition error rate of 5.1% [23]. The deep convolutional neural network uses structures such as weight sharing to construct spatial structure relationships, thereby reducing the number of parameters required for model generation, thereby improving the efficiency of backpropagation during model training. A general deep convolutional neural network consists of multiple convolutional layers, and there are usually two operations in each convolutional layer.

1. Training stage: the training set that has been marked with emotion polarity is subjected to text preprocessing, feature selection, and feature weight calculation and then input into the classifier for training. The purpose of this stage is to enable the classifier to correctly identify the target after training. The text is categorized.
2. Testing stage: preprocess the test set texts with marked emotional polarity, calculate feature weights, and input them into the trained classifier. By comparing the classification results with their corresponding marked emotional polarities, the performance of the device is evaluated.

After completing the above process, the classifier with good classification performance can be used in the classification task of sentiment text in the same field as the training test set. It can be seen from the above two stages that the text sentiment analysis method based on machine learning mainly includes three parts: text preprocessing, feature extraction, and classification algorithms.

### 3.1. Text Preprocessing

Under normal circumstances, the text obtained by data collection often contains a lot of noise, and direct analysis of it will affect the accuracy of sentiment analysis. The main processing object of text sentiment analysis is standardized and effective text information. Therefore, after obtaining the text, the first step is to remove invalid information and standardize text expression. The main steps include the following:

1. Use multiple different convolution kernels to filter the image and extract local features. For each convolution kernel, a new 2-dimensional image will be output from the input traditional pattern image.
2. The new traditional pattern images processed by different convolution kernels, through linear or nonlinear activation function processing, are mapped to a new feature space to form a new feature image set.
3. The newly formed feature traditional pattern image collection is down-sampling (i.e., pooling in the usual sense). This operation can effectively retain task-related features, remove irrelevant features, and improve the model's performance and normalization ability.

### 3.2. Convolution

Convolution operation refers to starting from the upper left corner of the traditional pattern image and opening an active window with the same size as the template. The window image and the template pixels are multiplied and then added, and the calculation result is used to replace the pixel in the center of the window. Then, the active window is moved one column to the right, and the same operation is performed. By analogy, from left to right, from top to bottom, you can get a new traditional pattern image. Its continuous definition is as follows:

(1) $$\begin{array}{c} \min_{G}\max\left(f*g\right)\left(x\right)=\int_{-\infty }^{+\infty }f\left(\tau \right)g\left(x-\tau \right)\text{d}\tau . \end{array}$$

**Figure 1:**
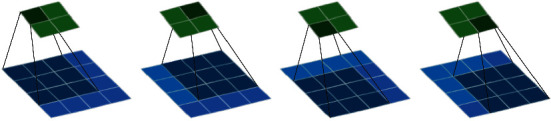
Schematic diagram of convolution budget.

Among them, *f* and *g* are two integrable functions on the set of real numbers. The schematic diagram is shown in Figure 1.

The figure shows the convolution operation on a 4 × 4 traditional pattern image. The size of the convolution kernel is 3 × 3, the convolution step size is 1 × 1, and the final convolution result is a 2 × 2 matrix.

#### 3.2.1. Dilated Convolution

**Figure 2:**
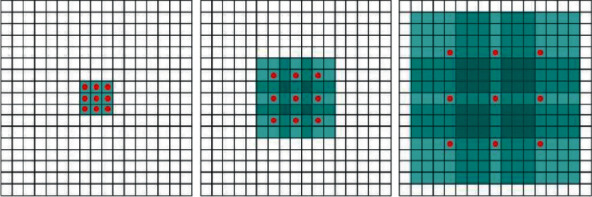
Schematic diagram of expanded convolution operation.

Dilated convolution is a convolution idea that is proposed for the traditional pattern image downsampling in the convolution, which will cause the traditional pattern image to have reduced resolution and lose information. For general convolution, the sampling position of the convolution kernel is expanded to increase the receptive field. For example, for a 3 × 3 convolution kernel with an expansion rate of 2, the receptive field range becomes 5 × 5. Compared with the original 3 × 3 receptive field, the receptive field is increased by 25/9 times, as shown in Figure 2.

As can be seen from the figure, the expanded convolution can effectively increase the receptive field range of the convolution kernel without increasing the amount of parameters, which is beneficial to the model to extract the features of the points in the relatively far distance distribution.

#### 3.2.2. Transposed Convolution

**Figure 3:**
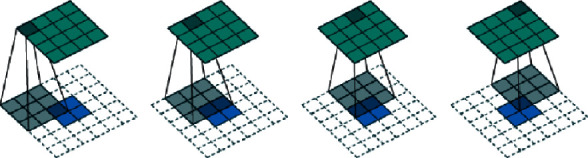
Schematic diagram of transposed convolution operation.

Transposed convolution is another operation based on convolution, which can restore the convolution result to the original traditional pattern image input before convolution. The calculation process is shown in Figure 3.

The convolution kernel of the transposed convolution uses the transposition of the general convolution kernel, so as to ensure that the position information between each element on the feature map remains unchanged. This is conducive to learning the feature relationship on the feature map.

#### 3.2.3. Application of Deep Convolutional Neural Network

**Figure 4:**
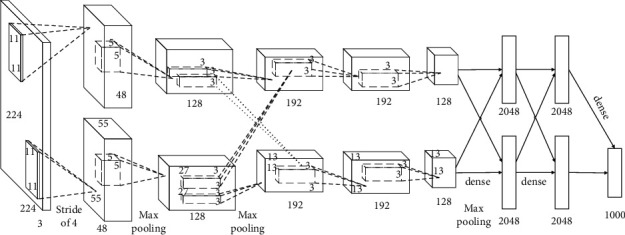
Deep convolutional neural network structure.

The deep convolutional neural network based on convolution operation is mainly used to extract and learn feature information in traditional pattern images, and the learned feature information is used to perform traditional pattern image classification tasks. The deep convolutional neural network based on convolution operation consists of 5 convolutional layers and 3 fully connected layers, and its network structure is shown in Figure 4.

In the deep convolutional neural network structure, the number of parameters used by the first 5 convolutional layers only accounts for about 5% of the total number of parameters of the model, but it can greatly improve the overall performance of the model. Reducing the convolution of any layer will lead to a reduction in the overall effect of the model. It can be seen that the deep convolutional neural network structure model based on convolution can effectively improve the efficiency and effectiveness of feature extraction.

**Figure 5:**
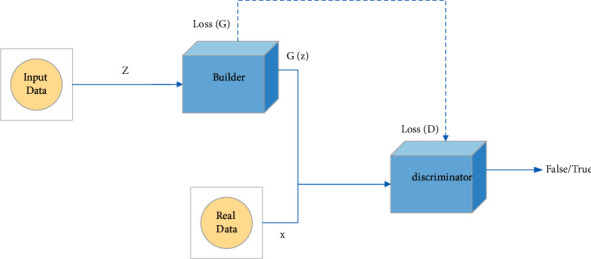
The training framework for generating a confrontation network (GAN).

The generative adversarial network (GAN) is an unsupervised machine learning network based on the 0-1 game theory. GAN usually consists of two networks with different functions: one is the generative network (GN), and the other is the discriminative network (DN). The generating network is used to generate candidate solutions for the task, and the discriminating network is used to determine whether these candidate solutions meet the requirements. Generally speaking, the generative network learns how to project the feature information in the hidden layer into the real data distribution. At the same time, the discriminative network learns to distinguish the data distribution produced by the generative network and the data distribution produced by the real data. The training task of generating the network is to increase the error rate of discriminating the network to determine the source of the data distribution. The GAN model can be usually expressed by the following formula:

(2) $$\begin{array}{c} \min_{G}\max_{D}V\left(D,G\right)=E_{x∼P\text{data}\left(x\right)}\left[\log\;D\left(x\right)\right]+E_{z∼Pz\left(x\right)}\left[\log\left(1-D\left(G\left(z\right)\right)\right)\right], \end{array}$$

where *D* is the discriminant network, *G* is the generating network, *V*(*D*, *G*) is the value function, *x*∼*P*data (*x*) means that x obeys the distribution of real data *P*data (*x*), and *z*∼*Pz* (*x*) means *z* obeys the random distribution *Pz* (*x*). It can be seen from the above formula that we only need to maximize the result of discriminating network *D* and minimize the result of generating network *G* to form an adversarial neural network, as shown in Figure 5.

## 4. Construction of Deep Neural Network in Ancient Chinese Pattern Repair Model

This article will present an ancient traditional pattern image restoration model based on the combination of deep convolutional neural network and adversarial neural network. The model combines multiple convolutional layers, transposed convolutional layers, and expanded convolutional layers to achieve the extraction and use of feature data of ancient traditional pattern images and finally realize the restoration of ancient traditional pattern images. To be able to effectively extract the feature information of the ancient traditional pattern image and understand the content information of the ancient traditional pattern image, the model in this paper uses a deep neural network to learn the features in the image in an unsupervised manner and use them for image restoration.

For the restoration of the ancient traditional pattern image, it is mainly aimed at the restoration of the geometric structure information and pattern information of the missing area. Since there is often a big difference between the geometric structure information and texture information of the missing region, this paper presents a two-stage generation network structure. Through the rough repair phase and the detailed repair phase, reasonable repair of the geometric structure information and pattern information of the missing area is realized. In addition, because the complexity of the pattern information often exceeds the geometric structure information, a large amount of feature information is required for reference. Therefore, this paper presents a method that uses multiple feature matching and comprehensively considers the restoration of the pattern information in the missing area.

**Figure 6:**
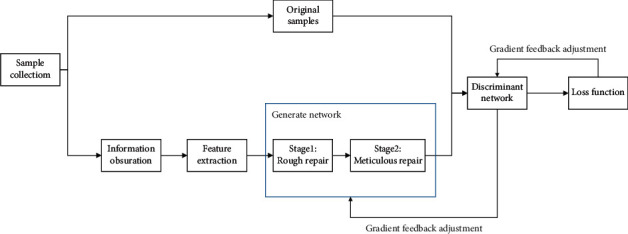
The structure of traditional pattern repair model.

In this paper, the ancient traditional pattern image restoration model based on deep neural network is mainly composed of five parts, which are information masking, feature extraction, generating network, discriminating network, and loss function. These parts each realize different functions, are relatively independent of each other but influence each other, and finally build the image restoration model of ancient traditional patterns given in this article. The model structure is shown in Figure 6.

### 4.1. Pattern Feature Extraction

**Figure 7:**
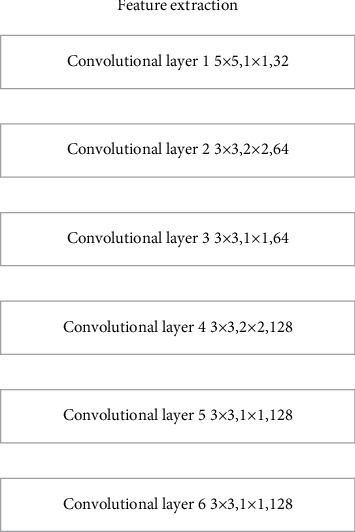
Feature extraction structure.

In order to effectively extract the remaining part of the feature information from the missing ancient traditional pattern image information, the feature extraction part implements the feature extraction of the ancient traditional pattern image information through a feature extraction network constructed by a 6-layer convolutional layer. In the feature extraction network constructed by the 6-layer convolutional layer, the convolution kernels of different sizes, the number of output channels, and the step size of the convolution kernel are used to improve the diversity of the features extracted by the feature extraction part. In order to be able to extract diversified feature information, this article draws on the experience of predecessors in designing deep learning models. First, a 5 × 5 size convolution kernel is used to extract more pixel feature information, and then a smaller 3 × 3 size volume is used. The product core screens many features. In this paper, through different convolution kernel step lengths, the convolution operation can have different convolution fields of view, so that a variety of different feature information can be extracted. Its structure is shown in Figure 7.

Since there is often a big difference between the geometric structure information and texture information of the missing region, this paper uses a two-stage generative network structure.Through the rough restoration phase and the detailed restoration phase, reasonable restoration of the geometric structure information and texture information of the missing area is realized.

#### 4.1.1. Rough Repair Stage

**Figure 8:**
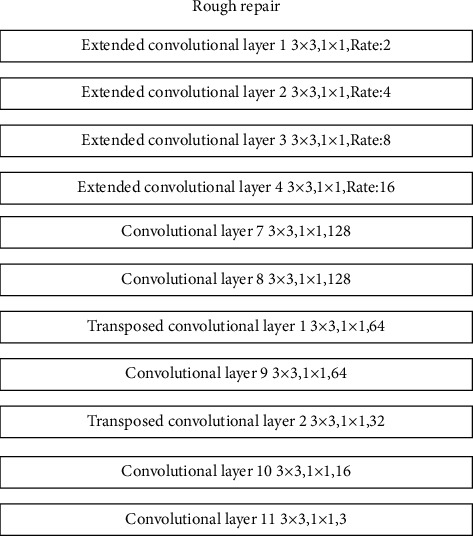
Rough repair structure.

In the rough restoration stage, the rougher restoration of missing information is mainly achieved by using multiple convolutional layers, expanded convolutional layers, and transposed convolutional layers. In the rough repair stage, a total of 4 dilated convolutional layers with different expansion rates, 5 convolutional layers with different output channels, and two transposed convolutional layers are used. The structure is shown in Figure 8.

In the subsequent 7-layer structure of the rough repair stage, the two-layer transposed convolutional layer and the five-layer convolutional layer are used to fill in the missing information. Finally, through the two-layer convolutional layer, the multiple feature information matrix repaired by the 2-layer transposed convolution can be converted into the three-layer ancient traditional pattern image information matrix, which realizes the rougher repair work for the missing part of the information.

#### 4.1.2. Detailed Repair Stage

**Figure 9:**
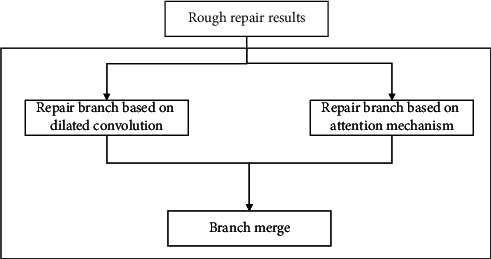
Detailed repair structure.

The multifeature comprehensive repair method generates repair information through two different information repair branches according to the repair results of the rough stage, then comprehensively considers the results of the different branches, and finally produces the repair results. The detailed repair stage is divided into three parts in total, which are the repair branch based on convolution, the repair branch based on the attention mechanism, and the branch merging. Its structure is shown in Figure 9.

**Figure 10:**
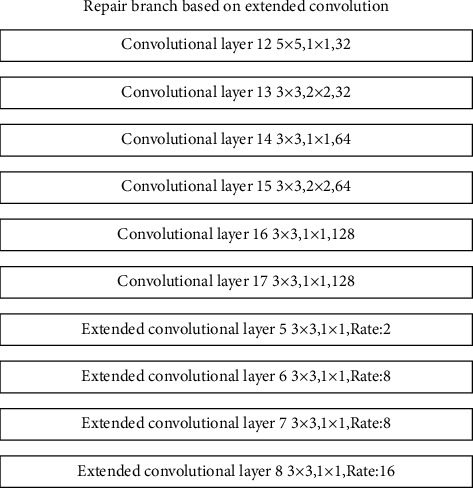
Repair branch structure based on dilated convolution.

The repair branch based on dilated convolution consists of 6 layers of convolution and 4 layers of dilated convolution, through different convolution layers with different sizes of convolution kernels, convolution step size and number of convolution kernels, and different dilated convolution layers. The expansion rate realizes the extraction of the feature information of the rough repair result. Its structure is shown in Figure 10.

**Figure 11:**
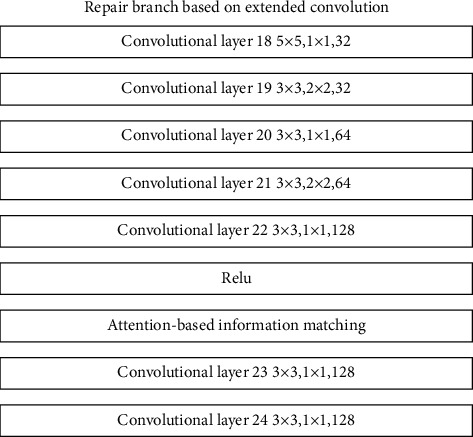
Repair branch structure based on attention mechanism.

The repair branch based on dilated convolution uses different dilated convolution rates in the last four layers of the branch, so as to realize the screening of feature information with relatively close positions. Through this branch, the model can extract features of peripheral regions that are relatively close to the features of the missing locale. At the same time, through the ReLU activation function, the more important feature information is filtered and input into the information matching structure based on the attention mechanism. The information matching structure based on the attention mechanism screens out the feature information that is far away from the missing area feature information but has a relatively large correlation. The repair branch structure based on the attention mechanism is shown in Figure 11.

**Figure 12:**
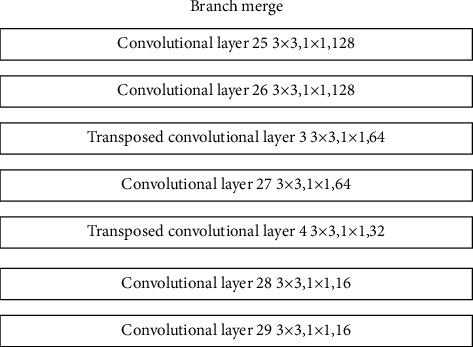
Branch merging structure.

In order to better complete the restoration of the image, it is necessary to rationally merge the results of the two different branches to produce the final detailed restoration result. For this reason, the branch merging part uses 5-layer convolution and 2-layer expansion convolution to achieve the final merging of the results of different repair branches. Its structure is shown in Figure 12.

**Figure 13:**
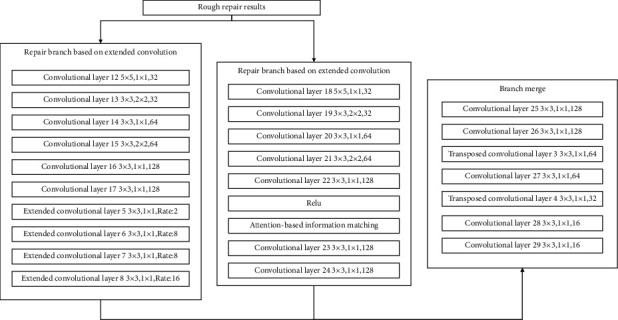
The overall structure of the detailed repair stage.

The overall structure of the detailed repair part is shown in Figure 13.

### 4.2. Discriminating Network

**Figure 14:**
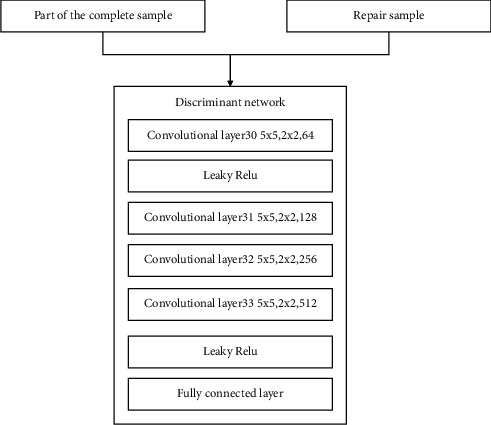
Discriminant network structure.

In the previous section, the network model generated in the model was introduced. This section introduces the discriminant network constructed by the multilayer convolutional layer, the nonlinear function activation layer, and the fully connected layer. The discriminant network part is composed of 4-layer convolutional structure, 4 Leaky ReLU activation function layers, and a fully connected layer. Its structure is shown in Figure 14.

The fully connected layer connects the feature information output by the last layer of Leaky ReLU and uses this feature information to classify the ancient traditional pattern image in two ways to determine whether it is an ancient traditional pattern image that has been partially repaired with missing information or an ancient traditional pattern image with complete original information. Thus, a complete confrontation neural network structure is constructed.

### 4.3. Loss Function

According to the classification results of the ancient traditional pattern image and the original ancient traditional pattern image obtained by the above model, the loss function is constructed. In this article, cross entropy is used as a loss function to evaluate model errors. The formula is shown in

(3) $$\begin{array}{c} \text{Loss}=-y\;\log\left(y^{*}\right)-\left(1-y\right)\log\left(1-y^{*}\right). \end{array}$$

Among them, Loss is the model loss error, *y* is the ratio of the ancient traditional pattern image with complete original information in the input data, and *y*^*∗*^ is the ratio of the original image in the discrimination result.

## 5. Model Application and Result Analysis

The model given in this topic has been tested and compared with other models on the same data set. This section will introduce the related measurement standards of image filling results. The data set used in the experiment is part of the Qinghai traditional embroidery image data set. The experimental results show that the method presented in this paper has better measurement indicators than the sample-based traditional embroidery image restoration method in Qinghai and the deep learning image restoration model, and the restoration results are more reasonable.

### 5.1. Experimental Environment and Data Set

**Figure 15:**
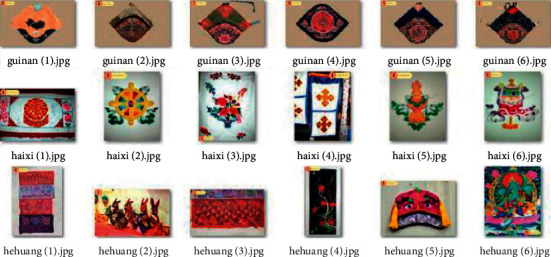
Part of the data set of traditional images of Qinghai embroidery.

Deep convolutional neural networks require a large amount of ancient pattern image data for training, and having sufficient and abundant ancient pattern image data can effectively prevent overfitting. The ancient pattern image data used in this article are all from the Qinghai Provincial Intangible Cultural Research Base. Before the edge extraction of the ancient pattern image, the original data set needs to be preprocessed through, for example, ancient pattern image translation, scaling, and cropping. The Qinghai embroidery image data set includes Tu nationality pan embroidery, Guinan Tibetan embroidery, Haixi Mongol, Hehuang embroidery, and Huangzhong pile embroidery with a total of 2000 pictures. In the experimental training phase, 2000 ancient pattern pictures can be divided into training set and test set, the ratio of the training set to the test set is 8 : 2, the training set consists of 1600 sheets, and the test set consists of 400 sheets, before the neural network training. It is necessary to adjust the size of the ancient pattern image to a smaller uniform specification to prevent the image attribute from being too large to affect the training process. Part of the data set of Qinghai embroidery images is shown in Figure 15.

### 5.2. Evaluation Index

In order to accurately reflect the results of different models after repairing the same ancient pattern image, this article uses multiple evaluation indicators to measure the performance differences of each model. The evaluation indicators used are L1 loss, L2 loss, peak signal-to-noise ratio (PSNR), and total variation loss (TV loss). L1 loss is defined as follows:

(4) $$\begin{array}{c} \text{L}1\text{ loss}=\frac{\sum \left|x^{f}-x^{r}\right|}{\sum_{i\in w}x_{i}^{f}}, \end{array}$$

where *x*^*f*^ represents the pixel information after the missing area is repaired, *x*^*r*^ represents the original pixel information of the missing area, and *w* represents the area to be repaired. The smaller the L1 loss value is, the closer the restored pixel information is to the original pixel information. The L2 loss is defined as follows:

(5) $$\begin{array}{c} \text{L}2\text{ loss}=\frac{\sum {\left(x^{f}-x^{r}\right)}^{2}}{\sum_{i\in w}{\left(x_{i}^{f}\right)}^{2}}, \end{array}$$

where *x*^*f*^ represents the pixel information after the missing area is repaired, *x*^*r*^ represents the original pixel information of the missing area, and *w* represents the area to be repaired. The smaller the L2 loss value is, the closer the restored pixel information is to the original pixel information. The peak signal-to-noise ratio is defined as follows:

(6) $$\begin{array}{c} \text{PSNR}=20*\log_{10}\left(\frac{{\text{MAX}}_{I}}{\sqrt{\text{MSE}}}\right),\\ \text{MSE}=\frac{1}{m*n}\overset{m-1}{\underset{i=0}{\sum }}\overset{n-1}{\underset{j=0}{\sum }}{\left(x_{i,j}^{f}-x_{i,j}^{r}\right)}^{2}. \end{array}$$

Among them, *x*_*i*,*j*_^*f*^ represents the pixel information after the missing area is repaired; *x*_*i*,*j*_^*r*^ represents the original pixel information of the missing area; MAX_*I*_ represents the maximum pixel value of the image; and *m*, *n* represent the horizontal position information and vertical position information of the pixels in the missing area. The larger the PSNR value is, the more diverse the pixel information after the restoration is reflected. The total variable loss is defined as follows:

(7) $$\begin{array}{c} \text{TV loss}=\frac{\left|{\text{VT}}^{f}-{\text{VT}}^{r}\right|}{{\text{VT}}^{r}},\\ {\text{VT}}^{f}=\underset{i,j}{\sum }{\left({\left(x_{i,j+1}^{f}-x_{i,j}^{f}\right)}^{2}+{\left(x_{i+1,j}^{f}-x_{i,j}^{f}\right)}^{2}\right)}^{1.25},\\ {\text{VT}}^{r}=\underset{i,j}{\sum }{\left({\left(x_{i,j+1}^{r}-x_{i,j}^{r}\right)}^{2}+{\left(x_{i+1,j}^{r}-x_{i,j}^{r}\right)}^{2}\right)}^{1.25}, \end{array}$$

where VT^*f*^ represents the total variation value of the missing area after repair, VT^*r*^ represents the original total variation value of the missing area, *x*_*i*,*j*_^*f*^ represents the coordinate of the missing area after repair (*i*, *j*), and *x*_*i*,*j*_^*r*^ represents the pixel information of the missing area whose original coordinates are (*i*, *j*). The smaller the VT loss, the stronger the correlation between pixels.

### 5.3. Related Methods

In order to correctly measure the performance of the image restoration model given in this article, in this section, two ancient pattern image restoration methods will be introduced.

Method 1 is a proposed image restoration method based on sample blocks. This method uses the local correlation of the image to perform image repair; that is, if the sample block B that matches the sample block A of the area to be repaired is found in the original image, the area around the sample block B may also be similar to the adjacent area of the sample block A.

**Figure 16:**
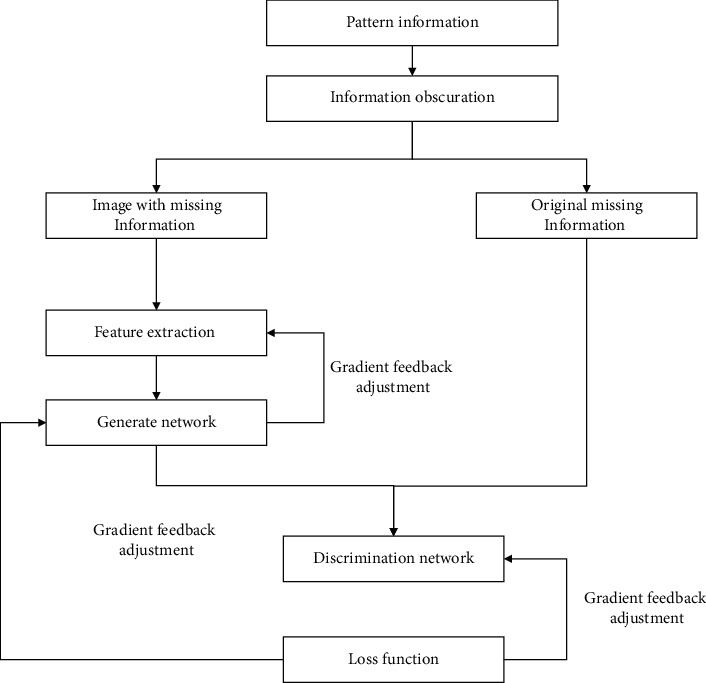
Method 2: results.

Method 2 is a traditional pattern image restoration method based on deep learning. This method is similar to the method of deep convolutional neural network in this article and also uses methods based on deep convolutional neural network and anti-neural network for traditional pattern image restoration. The method consists of four parts, namely, information masking, feature extraction, generating network, and discriminant network. Its structure is shown in Figure 16.

**Figure 17:**
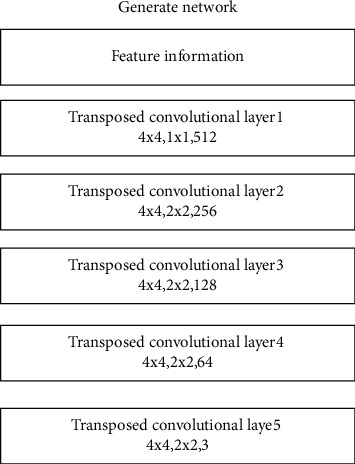
Method 2: generating network structure.

In method 2, the information masking part is the same as the method given in this paper, and the AlexNet network structure is used in the feature extraction part. The composition of the generating network and discriminant network of method 2 is relatively simple. The generation network of method 2 realizes the generation of missing area information through multilayer transposed convolutional layers. The discriminant network of method 2 realizes the classification of traditional pattern images through a multilayer convolutional layer and a fully connected layer. The generating network structure of method 2 is shown in Figure 17.

**Figure 18:**
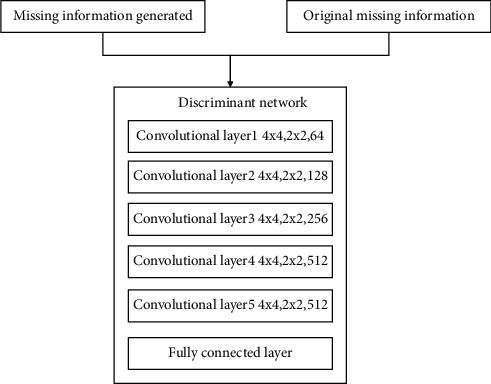
Method 2: discriminating network structure.

The discriminant network structure of method 2 is shown in Figure 18.

### 5.4. Experimental Results and Conclusions

In this section, method 1, method 2, and the method given in this article will be experimented on the Qinghai traditional embroidery image data set, and the experimental results will be provided.

The comparison results of image restoration on part of the Qinghai traditional embroidery image data set are shown in Table 1.

Table 1 shows that the method in this paper has better performance than methods 1 and 2 in terms of L1 loss, L2 loss, PSNR, and VT loss indicators. Method 1, method 2, and the method in this paper perform image restoration on some traditional pattern images on the Qinghai traditional embroidery image data set, and the results are as follows.

**Figure 19:**
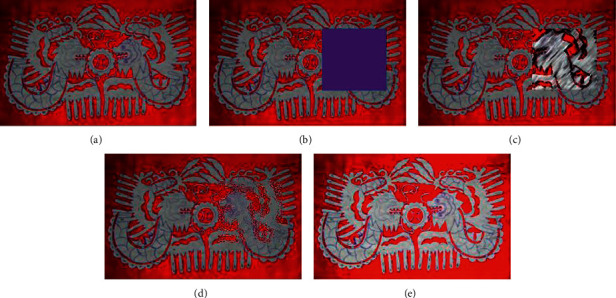
Comparison of repair results of different methods. (a) Original input image. (b) Masked image. (c) Method 1 repair result. (d) Method 2 repair result. (e) The repair result of the method in this article.

**Figure 20:**
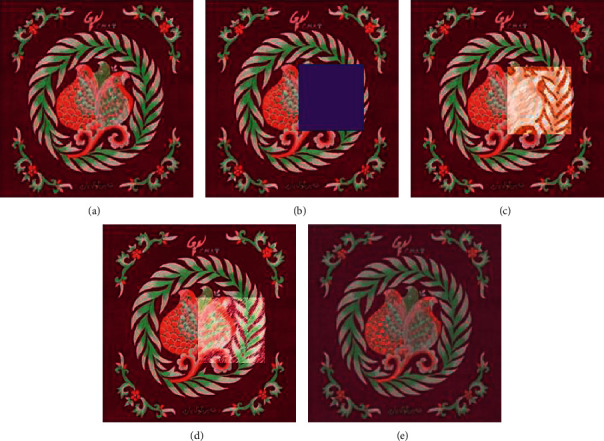
Comparison of repair results of different methods. (a) Original input image. (b) Masked image. (c) Method 1 repair result. (d) Method 2 repair result. (e) The repair result of the method in this article.

**Figure 21:**
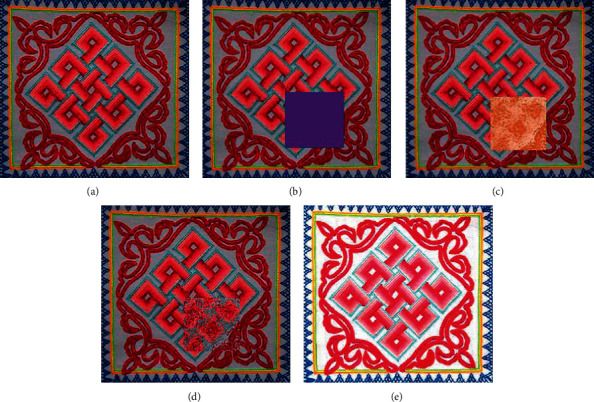
Comparison of repair results of different methods. (a) Original input image. (b) Masked image. (c) Method 1 repair result. (d) Method 2 repair result. (e) The repair result of the method in this article.

In Figures 19–21, (a) shows the traditional pattern images from the Qinghai traditional embroidery image data set, (b) is the traditional pattern image after the masking process, (c) is the traditional pattern image result after method 1 repair, (d) is the repaired image result of method 2, and (e) is the repaired image result of the method in this paper.

In Figures 19–21, the traditional pattern images that need to be repaired are all information masking for the central part of the image pattern, concealing most of the information in the image. From the results of restoration, method 1 has the worst restoration effect. The color of the restoration area is greatly affected by irrelevant background colors. The geometric features and texture information of the traditional pattern image after restoration are unreasonable. The restoration effect of method 2 is the second. The geometric information of the restored image is basically in line with people's perception of traditional patterns, but the texture effect processed by method 2 is poor. The restoration result of the method in this paper is better than the other two methods, and it can learn the geometric structure and texture information of the pattern and use this information to perform a more reasonable image restoration based on the information around the missing area.

According to the experimental results on the Qinghai traditional embroidery image data set, the method in this paper has a better measurement index of the traditional pattern image restoration effect than other methods. Because the information in traditional embroidery images is relatively complex, method 1 is easily affected by the pixel information in the background of the traditional pattern image, and it is difficult to recover the geometric structure information and texture information of the missing parts. Method 2 can better repair the geometric structure information of the missing parts of traditional patterns, but the repaired texture information is poor. Compared with method 1 and method 2, the method in this paper has more reasonable geometric structure and pattern information in the repair result. Therefore, the model given in this paper can effectively construct a reasonable traditional pattern image structure and texture information of the missing area based on the information characteristics around the missing area of the traditional pattern image, so as to realize the information restoration of the missing area.

## 6. Conclusion

The field of traditional pattern image restoration mainly studies how to reasonably repair the geometric structure and texture information of the missing parts of traditional pattern images. According to different theoretical foundations, there are 5 different methods in the field of traditional image restoration. They are image restoration methods based on partial differential equations, image restoration methods based on texture, image restoration methods based on samples, hybrid image restoration methods, and image restoration methods based on deep learning. Each of these methods has its own characteristics, and their focus is different. This article mainly presents an image restoration method based on deep learning, which combines deep convolutional network and adversarial neural network to achieve the restoration of traditional pattern image information. The effectiveness of the method in this paper is verified by running on part of the Qinghai traditional embroidery image data set. The work done in this paper is summarized as follows:

1. In-depth study of the research status of traditional pattern image restoration with the application of deep neural networks is conducted.
2. A traditional pattern image restoration model based on the combination of deep convolutional network and adversarial neural network is given. The model realizes the restoration of traditional pattern images through a two-stage generation network composed of rough restoration and detailed restoration.
3. A multifeature restoration method is introduced to complete the meticulous restoration of traditional pattern images, comprehensively considering different feature information for restoration.
4. This article comprehensively compares the traditional pattern image restoration methods based on traditional pattern samples and deep learning with the method given in this article. From the analysis of the experimental results, the method in this paper has better evaluation indicators and image restoration effects.

## Figures and Tables

**Table 1:** The evaluation index of method 1, method 2, and the method of this article.

Method	L1 loss (%)	L2 loss (%)	PSNR	TV loss (%)
Method 1	17.7	4.6	14.31	29.2
Method 2	10.6	2.5	20.26	25.5
Method of this article	8.3	2.1	22.92	24.8

## Data Availability

The data set can be accessed upon request.
